# Predictive Genetic Variations in the Kynurenine Pathway for Interferon-α-Induced Depression in Patients with Hepatitis C Viral Infection

**DOI:** 10.3390/jpm11030192

**Published:** 2021-03-11

**Authors:** Szu-Wei Cheng, Jing-Xing Li, Daniel Tzu-Li Chen, Yu-Chuan Chien, Jane Pei-Chen Chang, Shih-Yi Huang, Piotr Galecki, Kuan-Pin Su

**Affiliations:** 1 College of Medicine, China Medical University, Taichung 404, Taiwan; swchengjames@gmail.com (S.-W.C.); u105001791@cmu.edu.tw (J.-X.L.); peko80@gmail.com (J.P.-C.C.); 2 Department of Psychiatry and Mind-Body Interface Laboratory (MBI-Lab), China Medical University Hospital, Taichung 404, Taiwan; rocket1025918@gmail.com (D.T.-L.C.); yuchuan0916@gmail.com (Y.-C.C.); 3 Graduate Institute of Biomedical Sciences, China Medical University, Taichung 404, Taiwan; 4 School of Chinese Medicine, China Medical University, Taichung 404, Taiwan; 5 School of Nutrition and Health Sciences, Taipei Medical University, Taipei 110, Taiwan; sihuang@tmu.edu.tw; 6 Department of Adult Psychiatry, Medical University of Lodz, 90 Lodz, Poland; galeckipiotr@wp.pl; 7 An-Nan Hospital, China Medical University, Tainan 709, Taiwan

**Keywords:** *AFMID*, interferon-α-induced depression, *KYNU*, kynurenine pathway

## Abstract

**Importance:** The high incidence of major depressive episodes during interferon-α (IFN-α) therapy is considered the most powerful supportive evidence for the inflammation theory of depression. As the kynurenine pathway plays an important role connecting inflammation and depression, it is plausible to investigate this pathway for predictive genetic markers for IFN-α-induced depression. **Methods:** In this prospective case-control study, we assessed 291 patients with chronic hepatitis C viral infection taking IFN-α therapy and analyzed the single nucleotide polymorphisms (SNPs) in genes in the kynurenine pathway. Our case group contained patients who developed IFN-α-induced depression during the treatment, and others were defined as the control group. Genomic DNA was extracted from leukocytes in the peripheral blood and analyzed by Affymetrix TWB array. We first tested allelic, dominant, and recessive models on each of our SNPs using Fisher’s exact test. We then conducted 5000 gene-wide max(T) permutations based on the best model of each SNP to provide strong gene-wide family-wise error rate control. Finally, we preformed logistic regression for the significant SNPs acquired in previous procedures, with sex and education level as covariates to build predictive models. Additional haplotype analyses were conducted with Haploview 4.2 to investigate the combining effect of multiple significant SNPs within a gene. **Results:** With sex and education level as covariates, rs8082252 (*p* = 0.0015, odds ratio = 2.716), rs8082142 (*p =* 0.0031, odds ratio = 2.499) in arylformamidase (*AFMID)*, and rs12477181 (*p =* 0.0004, odds ratio = 0.3478) in kynureninase (*KYNU)* were significant in logistic regression models with dominant modes of inheritance. Haplotype analyses showed the two significant SNPs in *AFMID* to be in the same haploblock and highly correlated (r^2^ = 0.99). There were two significant haplotypes (by the sequence of rs8082252, rs8082142): AT (χ2 = 7.734, *p =* 0.0054) and GC (χ2 = 6.874, *p =* 0.0087). **Conclusions:** This study provided supportive evidence of the involvement of the kynurenine pathway in IFN-α-induced depression. SNPs in this pathway were also predictive of this disease.

## 1. Introduction

Interferon-α (IFN-α), the former standard treatment for chronic hepatitis C viral (HCV) infection, predisposed patients to a significant risk of major depressive side effects (IFN-α-induced depression), with incidence rates from 23% to 45% [1]. Despite the heterogeneity of depression [2], inflammation is widely considered as an important pathogenic factor [3,4,5,6,7]. Intriguingly, in addition to the depressive side effects of IFN-α, our research team also found several associations between IFN-α-induced depression and depressive disorders [8,9,10]. Therefore, IFN-α-induced depression is suitable for investigating the role of inflammation in depression in humans. In spite of the presence of a variety of widely accepted animal models for depression [11], establishing a human depression model is technically difficult and ethically problematic. IFN-α-induced depression thus provides us a rare and precious chance to attain such a model [12]. It also facilitates the understanding of the connection between immunology and psychiatry.

Previous studies investigated various mechanisms of INF-α-induced psychiatric symptoms [13,14,15,16,17]. One of the predominant mechanisms of interest connecting inflammation and depression was the kynurenine (KYN) pathway, as enzymes in this pathway can be directly affected by INF-α and other proinflammatory cytokines [18]. Additionally, a recent study showed the level of the downstream metabolites of the KYN pathway predicted treatment outcomes of the antidepressant reboxetine with celecoxib [19], further highlighting the importance of this pathway and its clinical predictive value.

In spite of the essential role of the KYN pathway in inflammation-induced depression, no comprehensive investigation was conducted into the genetic variations of this pathway on IFN-α-induced depression. As the age of personalized medicine comes, genetic testing is becoming a powerful tool in clinical settings. The scope of studied predictors of diseases also expanded from sociodemographic factors [20,21] to genetic predispositions [22,23]. Within this context, our study focused on the genetic variations of the KYN pathway to provide possible markers (1) to predict depressive side effects of IFN-α therapy in treated patients and (2) for the screening for susceptibility of inflammation-induced depression in the general population.

## 2. Methods and Materials

In this prospective case-control study utilizing the candidate gene approach, we investigated the genetic markers in the KYN pathway that predict the occurrence of depressive side effects in IFN-α therapy. We used the same cohort and genomic data as in one of our accepted articles [24] with completely different candidates and analytic methods.

This project was approved by The Research Ethics Committee of China Medical University and Hospital. The project identification codes and dates of approval are: CMUH104-REC1-022, 29 April 2015; CMUH104-REC1-022(CR-1), 09 March 2016; CMUH104-REC1-022(CR-2), 10 March 2017; CMUH104-REC1-022(FR), 13 December 2017.

### 2.1. Patient Selection

The subjects in our study were adult patients who were diagnosed with chronic HCV infection by qualified hepatologists and went to IFN-α therapy. These subjects had no records of interventions for depression in the previous 8 weeks. Patients with records of psychiatric disorders, strong suicidal ideations, or substance use disorder were excluded from this research. Eventually, there were 327 patients enrolled in this study. 

To fulfill our ethical obligations, we provided our subjects with educational information on IFN-α-induced depression. When depressive side effects occurred, treatments including antidepressants and psychosocial interventions were also provided. Before enrollment, all the subjects provided signed informed consent.

### 2.2. Study Procedure

Patients received 6 months (24 weeks) of IFN-α therapy for chronic HCV infection and were scheduled for regular appointments. The therapy was: 1.5 μg of peg-interferon α-2b per kilogram of body weight, administered subcutaneously once a week; 1000–1200 mg of ribavirin, administered daily. Patients were assessed for psychiatric disorders by psychiatrists with the Mini-International Neuropsychiatric Interview [25] based on the diagnostic criteria from Diagnostic and Statistical Manual of Mental Disorders, fourth edition (DSM-IV) at weeks 0, 2, 4, 8, 12, 16, 20, and 24. Only major depressive episodes were qualified as “IFN-α-induced depression.” Our case group contained patients who developed IFN-α-induced depression during the treatment, and others were defined as the control group. Sociodemographic characteristics of our patients, including sex, age, education level, marital status, and a history of psychiatric and substance use disorders, were also recorded. At our patients’ medical visits at weeks 0, 8, and 24, we obtained venous blood samples from them in the morning between 08:30 and 09:30 after overnight fasting for genotyping.

### 2.3. Genotyping

Genomic DNA was extracted from leukocytes using the QIAamp DNA Blood Mini kit (Qiagen, Crawley, UK). Genotyping was performed using Affymetrix TWB array (Axiom Genome-Wide Array Plate, Thermo Fisher Scientific, Waltham, MA, USA) by the National Center for Genome Medicine, Academia Sinica, Taipei, Taiwan.

### 2.4. Data Quality Control (QC)

We utilized Plink v1.90b6.14 to perform a genome-wide-level QC. The QC for subjects and the QC for single nucleotide polymorphisms (SNPs) were both performed. We performed the QC for subjects first, which was an exclusion of subjects with problematic clinical data. After the exclusion, we performed a sex check. When there was a mismatch between the assigned and the chromosomal sex of our subjects, we sought further confirmation on their sex and only retained them if we had it. Eventually, 291 out of 327 subjects passed this procedure, of which 66 were cases, and 225 were controls. A summary statistic was performed at this point, which showed all subjects had calling rates above about 97%. No further exclusion was conducted due to the fairly good quality of our data. We then performed the QC for SNPs. The SNPs retained in our study had all the following characteristics: (1) genotyping rate ≥ 95%; (2) minor allele frequency ≥ 0.01; (3) followed Hardy–Weinberg Equilibrium in controls, as tested by Exact Test [26] with a significance level of 0.001.

### 2.5. Candidate Gene Selection

We utilized established pathways in Reactome (https://reactome.org/ accessed on 7 April 2020) for the selection of candidate genes. The entry “Tryptophan catabolism” (R-HSA-71240) was selected on 7 April 2020 with an additional gene *QPRT*. Eventually, there were 15 genes in our study. Details of these genes and SNPs are shown in Table 1 and Supplementary Table S1.

### 2.6. Statistical Analysis 

The software we used for statistical analyses was Plink v1.90b6.14, with Gplink v2.050 as its user interface. There were two steps in our analyses.

In the first step, we tested allelic, dominant, and recessive models on each of our SNPs using Fisher’s exact test (full-model association test). We then performed 5000 gene-wide max(T) permutations based on the most significant result of the three models to provide strong gene-wide family-wise error rate control, and to create empirical *p*-values. In this process, the best original result for each SNP was compared against the others.

In the second step, we did logistic regression for each of our significant SNPs. The best model obtained in the first step was considered as the mode of inheritance, and possible confounders were chosen as covariates.

## 3. Results

### 3.1. Full-Model Association Test

SNPs with an empirical *p*-value < 0.05 were considered significant. We found two significant SNPs in arylformamidase (*AFMID*) and one in kynureninase (*KYNU)*. Details are shown in Table 2. 

### 3.2. Logistic Regression

Significant SNPs were put into logistic regression to build predictive models. According to the results of full-model association tests, the modes of inheritance were all dominant. In order to choose appropriate covariates, the characteristics of our subjects, including marital status, sex, age, and education level, were analyzed. Under a significance level of 0.05, there are significant differences in the sex ratio (*p =* 0.003) and the education level (*p =* 0.030) between our cases and controls. They are thus our covariates. The difference in the sex ratio might be explained by a higher prevalence rate of HCV infection among men [27] and the susceptibility to depression among women [28]. Logistic regression showed significant results for all of the significant SNPs acquired in full-model association tests: rs8082252 (*p* = 0.0015, odds ratio = 2.716), rs8082142 (*p =* 0.0031, odds ratio = 2.499), and rs12477181 (*p =* 0.0004, odds ratio = 0.3478). As for covariates, sex was significant for all of our significant SNPs, while the education level was not.

To further assess the role of sex for these three significant SNPs in the logistic models, we built alternative models with sex as the only covariate and additional sex*SNP interaction. The modes of inheritance were also all dominant. Under a significance level of 0.05, only rs12477181 in *KYNU* demonstrated significant interaction (*p =* 0.0241), indicating sex as a possible moderator for this SNP.

### 3.3. Haplotype Analysis

As there were two significant SNPs in *AFMID*, we intended to investigate the combining effect of them. We employed Haploview 4.2 to conduct the haplotype association tests. Only these two SNPs were selected for analysis. Haploview showed them to be in the same haploblock under the definition by Gabriel et al. [29], and the r^2^ was 0.99. We obtained two significant haplotypes (by the sequence of rs8082252, rs8082142): AT (χ2 = 7.734, *p =* 0.0054), and GC (χ2 = 6.874, *p =* 0.0087).

## 4. Discussion

To the best of our knowledge, this is the first study to comprehensively investigate genetic variations in the KYN pathways for IFN-α-induced depression. As depression is a heterogeneous disease differently classified by different methods [30,31], the inflammation-induced cohort we used had relatively homogenous etiology and was suitable to investigate the association between inflammation and the KYN pathway.

The main findings of our study are several SNPs in *AFMID* and *KYNU* that have predictive values for IFN-α-induced depression. There are two major aspects to discuss from these results. 

The first major aspect involves the pathogenic mechanisms of IFN-α-induced depression. Our study further supports the involvement of the KYN pathway. As was shown in one recent meta-analysis [32], two KYN-related patterns are present in depression: (1) a shift of tryptophan metabolism from serotonin to the KYN pathway and (2) a preferential metabolism of KYN to neurotoxic quinolinic acid (QUIN). These results supported tryptophan depletion and neurotoxicity as the pathogenic mechanisms of depression. Traditionally, researchers studying depression focused on the monoamine hypothesis and the role of serotonin. Because the KYN pathway catabolizes tryptophan into KYN [33], tryptophan depletion was considered as one possible pathogenic factor. A clinical study which investigated the biopsychosocial effects of IFN-α treatment on treated patients also showed tryptophan depletion as one of the contributing factors to IFN-α-induced depression [34]. However, the induction of neurotoxic KYN metabolites during inflammation is also a possibility [35]. After KYN is formed, it is metabolized into neurotoxic QUIN in the central nervous system [36], and QUIN may affect N-methyl-D-aspartate receptors [37] and glutamate regulations by astrocytes [38,39], resulting in an overstimulation of the glutamatergic system, neuronal structural changes, and even excitotoxicity [40]. In fact, our recently accepted article found several genetic variations in ionotropic glutamate receptor pathways associated with IFN-α-induced depression, providing evidence for the above concepts. Additionally, a recent review [41] also pointed out the roles of glutamate receptors and IFN-α-induced reduction of dopamine release in anhedonia, which is a common symptom of depression. The interesting interplay between glutamate and dopamine in IFN-α-induced depression is a promising topic for future studies.

Intriguingly, although previous studies showed that indoleamine 2,3-dioxygenase (IDO) could be induced by IFN-α [42] as well as IL-1β [43] and TNF-α [44], which are the cytokines induced by IFN-α, we did not find significant SNPs in *IDO1* or *IDO2*. One previous study also rendered similar results [45]. As IDOs directly catalyze tryptophan into KYN and are not expressed in the brain, [46] they may be related more to global tryptophan levels and the tryptophan depletion hypothesis, rather than QUIN/glutamate excitotoxicity. A lack of significant SNPs in *IDO*s with positive findings in enzymes in the latter steps of the KYN pathway implied that tryptophan depletion might be less important in the pathogenesis of IFN-α-induced depression, compared to the effects of KYN metabolites. Additionally, an animal study showed IDO and kynureninase (*KYNU*) can be activated together by high mobility group box 1 (HMGB1) [47], implying mechanisms which regulate these enzymes in a coherent way. Thus, in spite of a lack of evidence of pro-inflammatory cytokines affecting *KYNU* directly, *KYNU* may be affected indirectly by induced IDO. This could explain the significant SNP in *KYNU*.

Another important metabolite of the KYN pathway related to depression is kynurenic acid (KYNA). Contrary to neurotoxic QUIN, it is considered to be neuroprotective [48]. The contradictory effects of these two metabolites on neurons are intriguing. As previous studies showed an upregulation of neurotoxic QUIN in the brain [49] and lower plasma concentration of neuroprotective KYNA [50] in depressed patients, it may be the relative levels of QUIN and KYNA that determine the overall effects of KYN metabolites on depression. A meta-analysis also found decreased KYNA and increased QUIN levels in antidepressant-free patients, [51] further supporting the above concepts. However, why the balance of QUIN and KYNA shifts during inflammation should be elaborated in order to provide clear insights on the pathogenesis of IFN-α-induced depression. We propose two possible explanations. 

The first one is a cell-type related hypothesis. After KYN is formed, it is metabolized, in different cells in the brain, into KYNA and QUIN. KYNA is mostly produced in astrocytes, while QUIN is mostly produced in microglia [36]. As inflammation imposes different effects on astrocytic [52] and microglial [53] physiology, the balance of KYNA and QUIN may thus be shifted. The second one is a genetics-related hypothesis. The formation of KYNA and QUIN from KYN involves different enzymes. For the formation of KYNA, only kynurenine aminotransferases (KATs) are involved, but for the formation of QUIN, kynurenine 3 monooxygenase (KMO), KYNU, and 3-hydroxyanthranilic acid 3,4-dioxygenase (3-HAO) are involved. During inflammation, when IDO is induced and excessive KYN is produced, the balance of KYNA and QUIN may be affected by the quantity, property, or catalytic efficiency of these enzymes. Thus, genetic variations in genes encoding these enzymes may have an impact on inflammation-induced depression. Indeed, we obtained one significant SNP in *KYNU* in both the full-model association test and the logistic model. Additionally, we obtained two nominally significant SNPs (rs10760581, *p =* 0.0280; rs941960, *p =* 0.0280) in *KYAT1* (which encodes Kynurenine Aminotransferase 1) in the full-model association tests with recessive modes of inheritance.

The second major aspect is the clinical aspect. Despite the fact that IFN-α ceased to be the treatment of choice for HCV in 2017, it is still provided as a treatment option for Kaposi’s sarcoma and cancers. Thus, it is of utmost importance that we develop genetic testing to assess risks of depressive side effects of IFN-α treatments for our patients. Our study provided such predictive genetic markers for this purpose. In a more general sense, if we consider IFN-α-induced depression to be a human model for inflammation-induced depression, these markers may represent the susceptibility of an individual to this disease. As for the treatment for inflammation-induced depression, inhibiting the KYN pathway should be considered as a therapeutic strategy, and related pharmacological agents can be considered for development.

In our logistic models, sex is a significant covariate for every significant SNP. Considering sex differences are well-recognized in psychiatric disorders [54], it is not a surprising result. The significant sex*SNP interaction for rs12477181 in *KYNU* implied sex as a possible moderator, indicating the effect of rs12477181 polymorphisms on depression differed between males and females. Considering that depression may be caused by the additive effects of multiple biopsychosocial factors, if one sex suffers from certain predispositions, the effects of some SNPs may be greater in this sex compared to the other, and there may be significant sex*SNP interactions. From the biological perspective, previous studies showed females had lower total tryptophan concentrations [55] and decreased levels of neuroprotective KYNA [56]. This may make females more susceptible to inflammation-induced depression. A small nucleotide substitution in *KYNU* and a little change of its property may thus have a larger impact in females compared to males, so the significant interaction is plausible.

There are limitations originated from the methodology of our study. Firstly, we could not possibly collect data on every sociodemographic factor that could have an impact on IFN-α-induced depression. As a result, there is a lack of data on some potentially important variables in this study. Secondly, when employing the candidate gene approach and utilizing the Bonferroni correction to control family-wise error rates, statistical power may be reduced due to high linkage disequilibrium (LD) within genes. The permutation technique was used to solve this issue because it is a widely accepted “gold standard” method in genetic association studies and avoids high Bonferroni penalty [57]. Thirdly, false positives are genuine concerns in genetic association studies. One way to deal with these concerns in studies employing the candidate gene approach is to minimize the number of candidates. As the KYN pathway is a relatively small pathway with only 15 genes, it was a suitable target for our study. Fourthly, we could not determine the functional roles of the significant SNPs in our study. We noticed that rs8082252 and rs8082142 are in introns. However, rs12477181 was in *KYNU* in GRCH37 (the assembly used in our data) but is now in the intergenic zone near the upstream border of *KYNU* in GRCH38. They may be functional through mechanisms like affecting alternative splicing, being promotors or enhances, or they may be in LD with functional SNPs. None of our significant SNPs were addressed by previous studies. They may be plausible targets for future studies.

## 5. Conclusions

This study identified several SNPs in the KYN pathway with predictive value for IFN-α-induced depression and provided supportive evidence for the involvement of the KYN pathway in the pathogenesis of this disease.

## Figures and Tables

**Table 1:** Candidate gene information.

Official Symbol	Product Protein	Location (Chr)	Length (bp)	Number of SNPs after QC
*AADAT*	Aminoadipate aminotransferase	4	31,450	7
*ACMSD*	Aminocarboxymuconate semialdehyde decarboxylase	2	63,610	10
*AFMID*	Arylformamidase	17	20,384	3
*HAAO*	3-hydroxyanthranilate 3,4-dioxygenase	2	25,524	7
*IDO1*	Indoleamine 2,3-dioxygenase 1	8	14,981	4
*IDO2*	Indoleamine 2,3-dioxygenase 2	8	81,436	35
*KMO*	Kynurenine 3-monooxygenase	1	63,625	34
*KYAT1*	Kynurenine aminotransferase 1	9	48,962	4
*KYAT3*	Kynurenine aminotransferase 3	1	57,187	5
*KYNU*	Kynureninase	2	293,388	67
*QPRT*	Quinolinate phosphoribosyltransferase	16	18,990	3
*SLC36A4*	Solute carrier family 36 member 4	11	50,245	3
*SLC3A2*	Solute carrier family 3 member 2	11	32,871	3
*SLC7A5*	Solute carrier family 7 member 5	16	39,471	6
*TDO2*	Tryptophan 2,3-dioxygenase	4	16,713	1

Abbreviations: bp: base pair; chr: chromosome; QC: quality control; SNP: single nucleotide polymorphism.

**Table 2:** Significant SNPs.

Gene	SNP	Model	Empirical *p*-Value	*p*-Value	Minor Allele	Major Allele
*AFMID*	rs8082252	Dominant	0.0052	0.0018	G	A
	rs8082142	Dominant	0.0122	0.0032	C	T
*KYNU*	rs12477181	Dominant	0.0124	0.0002	T	G

Abbreviations: SNP: single nucleotide polymorphism. Empirical *p*-value means the *p*-values obtained with the permutation technique. *p*-value means the *p*-values obtained in the original Fisher’s exact tests.

## Data Availability

The data are not publicly available due to privacy issues.

## Supplementary Materials

The following are available online at https://www.mdpi.com/2075-4426/11/3/192/s1, Supplementary Table S1: SNP Information.
