# Supplementary material for: Predictive Genetic Variations in the Kynurenine Pathway for Interferon-α-Induced Depression in Patients with Hepatitis C Viral Infection
Source: J Pers Med. 2021 Mar 11;11(3):192. doi: 10.3390/jpm11030192 (PMC7998192; doi:10.3390/jpm11030192)
Supplement: Supplementary file 1 [file jpm-11-00192-s001.pdf]

Supplementary Table S1. SNP Information

Gene	SNP
AADAT	rs3796526
	rs13145318
	rs963660
	rs56358114
	rs72702395
	rs62344720
	rs76766179
ACMSD	rs17322446
	rs1893396
	rs4954187
	rs1942052
	rs12622574
	rs3819121
	rs16831124
	rs7564746
	rs6711390
	rs2166480
AFMID	rs8082252
	rs8082142
	rs8070127
HAAO	rs2304661
	rs13016201
	rs12613026
	rs3816183
	rs12998043
	rs13026205
	rs72613891
IDO1	rs7820268
	rs7010461
	rs62512635
	rs3739319
IDO2	rs7846217

rs11777027
rs10504014
rs2543041
rs2955903
rs7016315
rs1362846
rs2955893
rs2543058
rs28631334
rs7820968
rs16888478
rs61123871
rs4321987
rs888444
rs1035280
rs11435188
rs16888382
rs11786337
rs7845003
rs3927941
rs56055654
rs12542808
rs28666749
rs6996031
rs79682491
rs60895952
rs2543073
rs1421215
rs10109853
rs2909333
rs7017498
rs10958579
rs2981155
rs57431511

KMO	rs1932440
	rs77742045
	rs61825639
	rs3765801
	rs4660103
	rs2050512
	rs2050515
	rs2050505
	rs3014573
	rs10926510
	rs3014572
	rs75172809
	rs1932439
	rs1932441
	rs4509566
	rs1335899
	rs4518887
	rs12731674
	rs6661244
	rs10926515
	rs11808201
	rs3007743
	rs6658805
	rs397960509
	rs3007736
	rs74443223
	rs3753214
	rs12139931
	rs9970943
	rs12030098
	rs685722
	rs10926523
	rs655970
	rs1053230

KYAT1	rs60239222
	rs10760581
	rs941960
	rs2417133
KYAT3	rs60436973
	rs1325923
	rs2765524
	rs7526419
	rs76764652
KYNU	rs12691655
	rs12477181
	rs4662298
	rs6736332
	rs6706157
	rs1490949
	rs164605
	rs11902906
	rs34794422
	rs11902056
	rs352889
	rs73961708
	rs9013
	rs1017995
	rs10198899
	rs10928161
	rs10928162
	rs10496933
	rs164735
	rs13388996
	rs966624
	rs16858506
	rs77649985
	rs76494682
	rs6712291

rs117660796
rs34435542
rs12470820
rs17190532
rs13027801
rs1017994
rs1866621
rs16855204
rs956594
rs62170127
rs2016775
rs1465839
rs7586571
rs1439876
rs1438266
rs17805075
rs6429990
rs16858223
rs11692509
rs3768853
rs13030111
rs9917201
rs351696
rs12997823
rs13024113
rs351685
rs351684
rs7561260
rs1866619
rs62169913
rs6429997
rs4609990
rs62169914
rs1371516

	rs34332680
	rs352873
	rs352887
	rs189526
	rs352922
	rs10496939
	rs6430001
	rs34550924
QPRT	rs12596308
	rs9923341
	rs2303255
SLC36A4	rs10830993
	rs10831001
	rs10831005
SLC3A2	rs10897300
	rs489381
	rs4726
SLC7A5	rs1060253
	rs747578
	rs11865049
	rs8052746
	rs67288628
	rs876985
TDO2	rs2878125

Abbreviations: SNP: single nucleotide polymorphism
